# Blue Light Induced Edible Mushroom (*Lentinula edodes*) Proteomic Analysis

**DOI:** 10.3390/jof6030127

**Published:** 2020-08-06

**Authors:** Youn Jin Park, Myoung Jun Jang

**Affiliations:** Department of Plant Resources, Kongju National University, Yesan Campus, Yesan-eup, Yesan-gun, Chungcheongnam-do 340-702, Korea; coma1052@snu.ac.kr

**Keywords:** *Lentinula edodes*, blue light, two-dimensional electrophoresis, real time PCR

## Abstract

Blue light is an important environmental factor that induces mushroom growth and morphological changes. In this study, after confirming the morphological difference between *Lentinula edodes* (LE) under blue light condition (BL) and lightless condition (LL), the increase and decrease in LE protein and the expression of RNA of each protein were confirmed under each condition. LE specimens grown in BL and LL were identified by 253 spots in BL through 2D electrophoresis and LC-MSMS analysis, and 22 types of proteins were identified. It was confirmed that 14 types of proteins showed reduced expression in BL compared to LL. On the other hand, eight kinds of proteins with increased expression in blue light compared to LL were identified. As a result of confirming the difference from the expression pattern in 2D electrophoresis through Quantitative Real-Time PCR, it was confirmed that the expression pattern of the two proteins showed a difference. Therefore, this study will be a key study on the changes in mushroom morphology induced by blue light and the proteins that induce it.

## 1. Introduction

*Lentinula edodes* (LE) is a mushroom belonging to Basidiomycota, Marasmiaceae and Lentinula, and its area and production are increasing in Northeast Asia [1]. LE mushrooms have a unique flavor and taste and are mainly used for food in Northeast Asia, and have been known to have functional effects such as hypertension and arteriosclerosis [2].

Light acts as an important environmental signal that regulates the growth and morphogenesis of organic matter and produces secondary metabolites. In plants, phytochromes, a red and far-red light receptor, and cryptochromes, a blue light receptor, are known to participate in the regulation of growth and morphogenesis. Recent studies have shown that red and blue photoreceptors are found in Fungi and that photoreactivity associated with somatic cell growth [3], spore formation [4], phototoropism [5], and circadian cycle [6,7] was confirmed.

Previous studies have identified protein changes at the mycelia level, and many studies have focused on changes in secondary metabolites. In this study, the expression patterns of all proteins were compared through 2D electrophoresis method to confirm the morphological characteristics of LE fruit bodies grown in blue light and lightless conditions and various protein patterns related to LE. Proteins that influence photoreaction and morphological properties were investigated through PCR method.

## 2. Material and Methods

### 2.1. LE Materials and Growth Condition

The LE strain, Sanjo701ho was purchased by Federation Forest Mushroom Research Center (FMRC) in Korea. The fruit body generation medium was 80% of oak sawdust (Alpine tree 1: Shingal tree 1) (w:w). It was adjusted to 65% moisture content to produce a 1-kg plastic bag. Blue light (BL) condition was 300 lux. The cultivation condition was maintained at 25 °C and 85% humidity to generate mature fruit body.

On the other hand, the other LE was incubated in the same cultivation conditions except with lightless conditions (LL) as a control mushroom. The pileus and stripes were separately harvested at 1, 3, 5 days after incubation at different light conditions and stored at −80 °C until used.

### 2.2. LE Sample Preparation and 2D Electophoresis

LE sample preparation and 2D Electrophoresis were performed as essentially described by Park et al. (2004). Grinded LE powder samples were suspended in 50 mM Tris buffer containing 7M urea, 2M thiourea, 4% (*w/v*) CHAPS, and 16 μL protease inhibitor cocktail (Roche Molecular Biochemicals, Indianapolis, IN, USA) was added. The LE contains lysates that were homogenized and centrifuged at 12,000× *g* for 15 min. After that fifty units of Benzonase (250 units/μL; Sigma, St. Louis, MO, USA) was added to the lysate mixture and 1ml aliquot stored at −80 °C until use after quantitation by the Bradford method (Bio-Rad, Hercules, CA, USA). For 2D Electrophoresis analysis, pH 3-10NL IPG strips (Amersham Biosciences) were rehydrated in swelling buffer. The protein lysates (500 μg/mL) rehydrated IPG strips using a Multiphor II apparatus (Amersham Biosciences) for a total of 57 kVh. The 2D Electrophoresis separation was carried on 12% (*v/v*) SDS-polyacrylamide gels. After that, following fixation of the gels for 1 h in a fixing solution of 40% (*v/v*) methanol containing 5% (*v/v*) phosphoric acid. The fixed gels were stained with Colloidal Coomassie Blue G-250 solution (ProteomeTech, South Korea) for 5 h. The gels were destained in 1% (*v/v*) acetic acid for 4 h and then imaged using a GS-710 imaging calibrated densitometer (Bio-Rad, Hercules, CA, USA).

Protein spot detection and 2D electrophoresis gel spot pattern matching were carried out using ImageMasterTM 2D Platinum Software (Amersham Biosciences).

### 2.3. Protein Spots with Trypsin and Extraction of Peptides

The experiment for in-gel digestion of protein spots from Coomassie Blue stained gels was performed as follows [8,9]. Protein spots were resected from stained gels. The gel pieces were cleaned for 1hr at room temperature in pH 7.8, 25 mM ammonium bicarbonate buffer, containing 50% (*v/v*) acetonitrile (ACN). The gel pieces were dehydrated in a SpeedVac(Thermo fisher) for 10 min and were rehydrated in 10 μL (20 ng/μL) of sequencing grade trypsin solution (Promega, WI, USA). After gel pieces were incubated in pH 7.8, 25 mM ammonium bicarbonate buffer, at 37 °C overnight, the trypsin-digested peptides were extracted with 5 μL of 0.5% TFA containing 50% (*v/v*) ACN for 40 min with gentle sonication. The extracted peptide solution was reduced to 1 μL in a vacuum centrifuge.

After that, the peptides solution was subjected to a desalting process using a reversed-phase column [10]. A GEloader tip (Eppendorf, Hamburg, Germany) was packed with Poros 20 R2 resin (PerSpective Biosystems, MA, USA). After an equilibration step with 5% (*v/v*) formic acid, the peptides solution was loaded on the column and washed. The peptides were eluted with α-cyano-4-hydroxycinnamic acid (CHCA) and dropped onto MALDI plate (96 × 2; Applied Biosystems, Forster city, CA, USA).

### 2.4. Proteins Identified by LC-MS/MS

The trypsin-digested peptides were separated and analyzed using reversed phase capillary HPLC directly coupled to LC-MS/MS [11]. Both a trapping and a resolving column were packed with C18 beads (5 μm in size, 300Å in pore size; Vydac, Hesperia, CA, USA) and placed in column line. Following this the peptides were bound to the trapping column for 10 min with acetonitrile containing formic acid, then the bound peptides were eluted with a 50-min gradient acetonitrile containing formic acid at a flow rate of 0.2 μL/min. For mass spectrometry, a full mass scan range was *m*/*z* = 450–2000 Da.

The individual MS/MS spcectra were carried out using the TurboSEQUEST software (Thermo Quest, San Jose, CA, USA). The generated peak list was used with the MSDB database and NCBI using the MASCOT program. (Available in Supplementary Materials).

### 2.5. Gene Onthology Analysis

Gene ontology categorization of the putative genes was performed according to the instructions of the Gene Ontology database (http://www.geneontology.org/, http://www.uniprot.org/, http://www.genome.jp/).

### 2.6. RNA Isolation

For identified gene expression profiles, total RNA was extracted from whole LE fruit body. Total RNA extractions were prepared from whole LE fruit body using RibospinTM^II^ Kit (Geneall Biotechnology, Seoul, Korea). Total LE RNA quality was measured by Agilent 2100 bioanalyzer using RNA 6000 Nanoship (Agilent Technologies, Amstelveen, The Netherlands). The RNA quantification was confirmed using ND-2000 spectrophotometer (Thermo Inc., Waltham, MA, USA).

### 2.7. Real-Time PCR Analysis

For qRT-PCR analysis, total LE RNA (1 μg) was converted into cDNA using a Power cDNA Synthesis Kit (iNtRON Biotechnology, Seoul, Korea). The cDNA synthesis process was started at 42 °C for 60 min, followed by incubating at 95 °C for 5 min to terminate cDNA synthesis reaction. The LE 18S (18S ribosomal protein) gene expression stability in LE served as housekeeping gene for normalization of the qRT-PCR [12]. After dilution cDNA, qRT-PCR was performed with Rotor-Gene Q 2plex HRM (Qiagen, Hilden, Germany) using the Rotor-Gene SYBR Green PCR Kit (Qiagen, Hilden, Germany). The PCR reaction condition was denaturation condition 95 °C for 10 min, annealing 40 cycles of 10 s at 95 °C, 15 s at an annealing temperature, 1 min at 72 °C. Real-time PCR experiments were conducted in three replications of each gene-specific primer for 2D electrophoresis-selected proteins. The relative gene expression was calculated using the 2^−ΔΔCt^ method compared to the LE sample with LL condition as control [13].

## 3. Results

### 3.1. Morphological Differentiation in BL and LL Condition of LE

**Figure 1:**
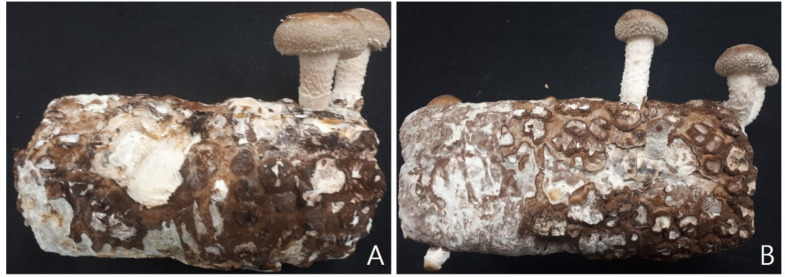
Fruit body of *Lentinula edodes* (LE) in Blue light (BL)and Lightless (LL) conditions. (**A**): BL, (**B**): LL.

In order to confirm the growth characteristics of LE-responsive growth, mushroom feet were applied in BL(A) and LL(B) conditions (Table 1, Figure 1). The results show that the pilus diameter is increased to 41.17 am in the BL condition and 28.42 bm in the LL condition and the pilus diameter increases in the BL condition. The thickness of the pilus diameter was 24.35 mm in BL and 23.82 mm in LL. On the other hand, the stipe length was 60.12a mm in LL condition and 40.75b mm in BL condition. Stipe thickness was increased to 29.45a mm at BL and 22.17b mm at LL condition. Since this apparent morphological differentiation was observed in LE, it was judged that the related protein was influential and the profile of whole protein was confirmed.

### 3.2. DE Analysis of the Fruit Body Protein Profile of LE

**Figure 2:**
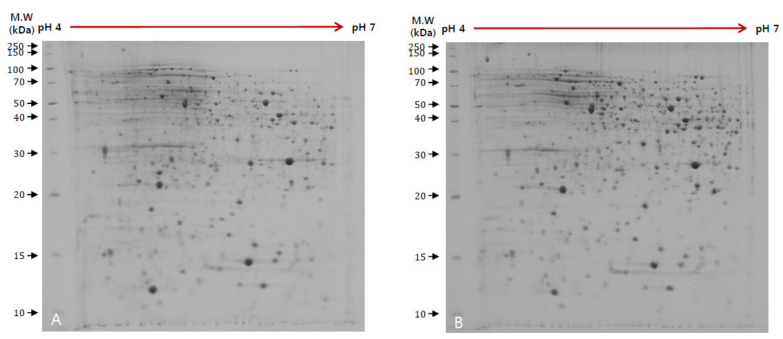
Representative 2D electrophoresis image of LE.; (**A**): Blue light (BL), (**B**): Lightless (LL).

Based on the morphological differentiation results of LE, protein expression pattern was confirmed by 2D electrophoresis (Figure 2). The increase and decrease of protein expression of 225 spots were confirmed (Table 2). Among them, 22 proteins were identified as up-regulated protein in BL condition and 16 kinds of protein as down-regulated protein. The protein with the highest down regulation in BL condition was confirmed by t-complex protein 1 (GAW01711.1), and the protein with down regulation was the second most abundant that decreased in the non-oxidative pentose phosphate pathway-related protein. The most up-regulated protein was phosphopyruvate hydratase (GAW03742.1), followed by testicular acid phosphatase like protein (GAW03420.1). To confirm the expression pattern of the protein identified in the above results, qRT-PCR was performed.

### 3.3. Validation of 2D Electrophoresis Data by qRT-PCR

**Figure 3:**
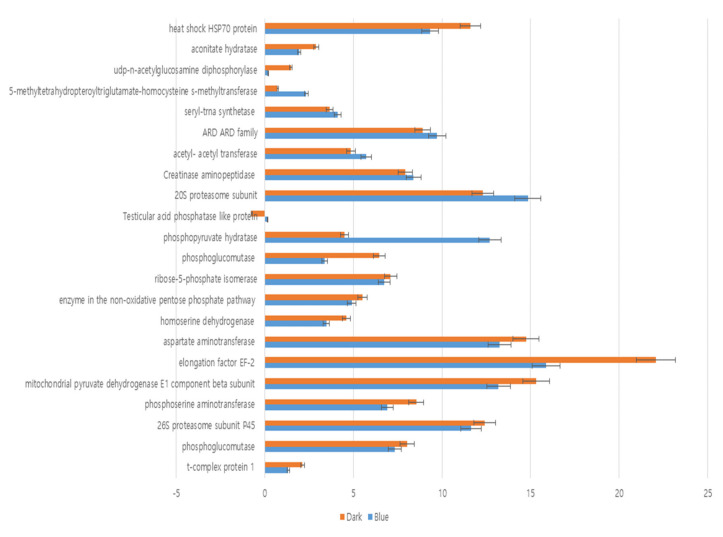
RT-qPCR analysis of 22 proteins in LE fruit body proteins.

The genes identified in 2D electrophoresis were analyzed by qRT-PCR. The results of 9 up-regulated proteins and 16 down-regulated analyzes in BL conditions were verified. To verify the expression levels, 18S RNA, a housekeeping gene, was used (Figure 3). The expression level of 14 genes down regulated in LL condition was the same as that of 2D electrophoresis analysis. The same expression pattern was also observed under the BL condition.

## 4. Discussion

Fungi species induce morphological changes depending on the light wavelength. These studies have remained at the mycelial stage until now. Studies on the mycelial stage were a clue to the morphological changes of fruiting bodies of actual mushroom cultivars. In this study, we have identified proteins that induce unknown morphological changes by inducing fruit body growth under BL and LL conditions by inoculating LE seeds into sawdust culture medium.

The morphological difference in LE was increased in the BL condition and the pilus diameter did not show any significant difference. Stipe length was longer in LL condition and stipe thickness was thicker in BL condition. It was confirmed that the morphological change was induced in the LE fruit body, which is an edible mushroom, under the BL condition. BL is also associated with circadian rhythms in *Neurospora crassa*, which increases the expression of light-responsive genes, thereby increasing the expression of the photoreceptor protein, a light-dependent signaling pathway [14,15].

The expression patterns of proteins were confirmed by 2D electrophoresis to identify proteins that induced morphological changes in the fruit bodies induced by BL. Phosphopyruvate hydratase, a protein up-regulated in BL, is known as 14-3-2 protein, 2-phosphoglycerate dehydratase or enolase, and catalyzes the conversion of 2-phosphoglycerate to phosphoenlypyruvate [16]. The expression of phosphopyruvate hydratase, which is involved in energy metabolism, in the BL condition is increased to Moscot score 730, suggesting that LE metabolism is active. In particular, 5-methyltetrahydropteroyltriglutamate-homocysteine s-methyltransferase, which is the second highest expression level, is known to be involved in methionine regeneration through methionine biosynthesis and methyltransfer [17], and is found in the fungi cell wall [18,19]. In order to confirm the expression level of up-regulated proteins in this BL condition, the result of qRT-PCR was also the same as the result of 2D electrophoresis. Thus, the expression of proteins affecting cell metabolism and cell wall was higher than that of LL condition. It is judged to induce a morphological change.

Enzymes in the non-oxidative pentose phosphate pathway, the most abundant expression level of down-regulated proteins in BL, were involved in the first step of the pentose phosphate pathway in *Escherichia coli* and catalyzed the conversion of ribose-5-phosphate to ribose-5 [20]. *Homosapiense* cells are mainly present in Cytosol [21]. In addition, the 26S proteasome subunit P45, which has a high expression level, binds to ATP as a ubiquitinated protein of ATP [22] and binds to ubiquitin protein ligase [23]. Down regulation of the expression levels of the above two proteins under BL conditions is considered to be an important consequence of the morphological change of LE, which is confirmed by qRT-PCR.

The results of this study suggest that expression of transcripts and protein expression is not a key prototype that directly induces morphological changes. However, expression of proteins other than genes revealed to be involved in fungi light response has been confirmed. In particular, the expression of the white collar complex (WC-1) [24], which has been identified as a gene involved in vital rhythm, life cycle, time, temperature, etc., was not observed in *Neurospora crassa.*

Therefore, the significance of this study is that it is the first report on proteins and transcriptomes related to photoreactivity of edible mushroom LE, and it is significant as a study that relates the expression of each protein and a related transcriptome. We will study the expression patterns between the identified proteins and the proteins that are influenced by the unknown mushroom’s optical signal transduction.

## Figures and Tables

**Table 1:** Pileus and stipe growth of LE.

	Pileus Diameter (mm)	Pileus Thickness (mm)	Stipe Length (mm)	Stipe Thickness (mm)
Blue light	45.17a	24.35a	40.75b	29.45a
Lightless	28.42b	23.82a	60.12a	22.17b

DMRT: Duncan’s Multiple Range Test. *p* > 0.5.

**Table 2:** Protein expression identified in LL versus BL.

Regulation	NCBI BLAST	Protein Name	Moscot Score
Down regulation	GAW04101.1	T-complex protein 1	389
	GAW00498.1	Phosphoglucomutase	260
	GAW02240.1	26S proteasome subunit P45	543
	GAW02323.1	Phosphoserine aminotransferase	398
	GAW07818.1	Mitochondrial pyruvate dehydrogenase E1 component beta subunit	350
	GAV98887.1	Elongation factor EF-2	487
	GAW06625.1	Aspartate aminotransferase	318
	GAW05175.1	Homoserine dehydrogenase	123
	GAW98631.1	Enzyme in the non-oxidative pentose phosphate pathway	782
	GAW00953.1	Ribose-5-phosphate isomerase	154
	GAW00498.1	Phosphoglucomutase	117
	GAW98802.1	Udp-n-acetylglucosamine diphosphorylase	191
	GAW06937.1	Aconitate hydratase	247
	GAW08468.1	Heat shock HSP70 protein	137
Up regulation	GAW03742.1	Phosphopyruvate hydratase	730
	GAW03420.1	Testicular acid phosphatase like protein	405
	GAV99150.1	20S proteasome subunit	281
	GAW07558.1	Creatinase aminopeptidase	359
	GAW01244.1	Acetyl- acetyl transferase	318
	GAW05495.1	ARD ARD family	176
	GAW03576.1	Seryl-trna synthetase	237
	GAW06001.1	5-methyltetrahydropteroyltriglutamate-homocysteine s-methyltransferase	592

qRT-PCR analysis of the selected protein profile of LE.

## Supplementary Materials

Supplementary materials are available online at https://www.mdpi.com/2309-608X/6/3/127/s1.
